# SCF increases cardiac stem cell migration through PI3K/AKT and MMP-2/-9 signaling

**DOI:** 10.3892/ijmm.2014.1773

**Published:** 2014-05-07

**Authors:** JUNLI GUO, WEI JIE, ZHIHUA SHEN, MENGSEN LI, YOULING LAN, YUEQIONG KONG, SHAOLI GUO, TIANFA LI, SHAOJIANG ZHENG

**Affiliations:** 1 Cardiovascular Institute of Affiliated Hospital, Hainan Medical College, Haikou 571199, P.R. China; 2 Department of Pathology, School of Basic Medicine Science, Guangdong Medical College, Zhanjiang 524023, P.R. China; 3 Hainan Provincial Key Laboratory of Carcinogenesis and Intervention, Hainan Medical College, Haikou 571199, P.R. China

**Keywords:** cardiac stem cells, stem cell factor (SCF)/c-Kit system, PI3K/AKT signaling, cell migration, matrix metalloproteinase-2, matrix metalloproteinase-9

## Abstract

The transplantation of cardiac stem cells (CSCs) is thought to be responsible for improving the performance of injured heart induced by myocardial infarction (MI). However, the mechanisms involved in the migration of activated CSCs post-MI remain to be clarified. In this study, CSCs were isolated from rat hearts and a cellular migration assay was performed using a 24-well Transwell system. Stem cell factor (SCF) induced CSC migration in a concentration-dependent manner, which could be blocked with an SCF antibody as well as a PI3K/AKT inhibitor, LY294002. Moreover, SCF induced the expression and activity of matrix metalloproteinase (MMP)-2 and MMP-9 in a concentration- and time-dependent manner, as measured by quantitative RT-PCR, western blot analysis and gelatin zymography. Results of western blot analysis revealed phosphorylated AKT was markedly increased in SCF-treated CSCs and that inhibition of SCF/c-Kit signaling or phospho-AKT activity significantly attenuated the SCF-induced expression of MMP-2 and MMP-9. Thus, our results showed that SCF partially mediated CSC migration via the activation of PI3K/AKT/MMP-2/-9 signaling.

## Introduction

Increasingly, ischemic heart diseases such as myocardial infarction (MI) are important causes of morbidity and mortality worldwide (1). Due to the loss of cardiomyocytes and formation of scar tissue after MI, cardiac function deteriorates and a series of fatal complications inevitably occur. Current treatment strategies based on stem cell transplantation for ischemic heart diseases are emerging, particularly those utilizing adult stem cells from heart tissues, termed cardiac stem cells (CSCs) (2–6). Phase I clinical trials such as SCIPIO (NCT00474461), ALCADIA (NCT00981006) and CADUCEUS (NCT00893360) involving heart-derived cells have been conducted (4). The feasibility, safety and effectiveness of injection of autologous heart-derived cells were assessed in these clinical trials with encouraging preliminary results, as evidenced by the reduction in myocardial scar mass or improvement in the left ventricular ejection fraction following cell treatment (7,8).

CSCs comprise a cluster of small cells located in a specific niche of the heart. Some subtypes of CSCs have been reported based on cell surface markers and cardiac location (9–12). In 2003, Beltrami *et al* (9) first reported that Lin^−^/c-Kit^+^ CSCs were detected in the adult rat heart, as located in the atria, apex and base-midregion of the ventricle. C-Kit^+^ CSCs are multipotent stem cells that can differentiate into myocardiocytes, smooth muscle cells and vascular epithelia cells under certain conditions. Findings of recent studies showed that c-Kit^+^ CSC transplantation improved the performance of heart tissue injured through coronary artery ligation (13,14). The results of the SCIPIO clinical trial also showed that transplantation of c-Kit^+^ CSCs enhanced the ejection fraction *in vivo* (7). Ellison *et al* reported that c-Kit^+^ CSCs are necessary and sufficient for functional cardiac regeneration and repair following myocardial damage (15). These reports highlight the viability and effectiveness of c-Kit^+^ CSC transplantation in myocardial regeneration.

Myocardium in peri-infarcted zones is in a state of stress post-MI, thus, several cardioprotective molecules including, but not limited to, PI3K, hypoxia-induced factor 1 (HIF1), NOTCH1 and stromal cell-derived factor (SDF), are upregulated (16–20). Previous results indicated that stem cell factor (SCF), a powerful stem cell chemokine, is upregulated in the cardiomyocytes of peri-infarcted zones (21), thus activating the chemokine signaling of the SCF/c-Kit axis. In this manner, c-Kit^+^ CSCs are migrated towards injured areas to fulfill critical roles in the process of myocardial regeneration. Endogenous c-Kit^+^ CSCs are located mainly in the niche of the atria, while most MI lesions clinically occur within the left ventricular because of left anterior descending (LAD) coronary artery disorders. Consequently, there is a large barrier that the chemoactivated c-Kit^+^ CSCs in atria must navigate when migrating towards injured zones within the left ventricular post-MI. Further knowledge regarding the mechanisms involved in the migration of activated c-Kit^+^ CSCs post-MI would therefore strengthen the evidence for CSCs transplantation in the treatment of MI.

PI3K/AKT signaling is known to be an important signal transduction cascade involved in cancer cell survival, apoptosis and motility (3). This type of signaling is crucial in stem cell biology. Activation of the PI3K/AKT pathway is crucial for VEGF-mediated c-Kit^+^ CSC migration *in vitro* and *in vivo* (22), and enhances cellular engraftment post-MI (23–25). However, the role of the PI3K/AKT pathway in SCF/c-Kit signaling-mediated CSC migration remains elusive. In the present investigation, we aimed to explore the crosstalk of SCF/c-Kit and PI3K signaling in the migration of c-Kit^+^ CSCs. Our results indicated that SCF-mediated c-Kit^+^ CSCs migration occurs at least partly via the activation of PI3K/AKT/matrix metalloproteinase (MMP)-2/-9 signaling.

## Materials and methods

### Isolation and culture of CSCs from adult rat hearts

CSCs were isolated by magnet-activated cell sorting (MACS) from the hearts of male Sprague-Dawley rats as described previously (13,21). Briefly, the heart was excised and the aorta was rapidly cannulated, followed by perfusion with Ca^2+^-free Tyrode solution for 10 min and then digestion with 0.5 mg/ml collagenase (Sigma, St. Louis, MO, USA) and 0.05 mg/ml trypsin (Difco, Kansas, MO, USA) at 37°C for 30 min. The heart tissue was sectioned and the resulting cell suspension was filtered with a strainer (Becton-Dickson, Franklin Lakes, NJ, USA). Cells were then incubated with a rabbit anti-c-Kit antibody (1:50; Santa Cruz Biotechnology, Inc., Texas, USA) and separated using immunomagnetic microbeads (Miltenyi Biotech, Bergish Gladbach, Germany). CSCs were then cultured in Dulbecco’s modified Eagle’s medium/Ham’s Nutrient Mixture F12 (1:1) (DMEM/F12) (Sigma-Aldrich) containing 15% fetal bovine serum (FBS) (Gibco, Carlsbad, CA, USA), 10 ng/ml basic fibroblast growth factor (bFGF), 20 ng/ml epidermal growth factor (EGF) (both from Sigma-Aldrich) and 2.5 μ/ml erythropoietin (EPO) (BioLegend, San Diego, CA, USA) at 37°C. After 28 days of culture, confluent CSCs were passaged.

### RNA isolation and quantitative RT-PCR (RT-qPCR)

Total RNA was extracted with TRIzol reagent (Invitrogen Life Technologies, Carlsbad, CA, USA). The total RNA (1 μg) was used as a template to generate cDNA by oligo(dT18) using the Fermentas RT System (cat. no. K1622; Thermo Fisher Scientific, Inc., Guangzhou, China). Primer pairs (5′-3′) used for PCR were synthesized by Sangon Biotech Co., Ltd. (Shanghai, China) and are shown in Table I. PCR products were separated by 1.5% agarose gel electrophoresis and visualized under UV using a gel documentation system (Bio-Rad, Hercules, CA, USA). RT-qPCR was conducted using the LightCycler480 instrument [Roche (China) Ltd., Shanghai, China] in a final volume of 20 μl, which included 10 μl SYBR-Green I PCR Master mix (Toyobo Co., Ltd., Osaka, Japan), 0.4 μl forward primer (10 μM), 0.4 μl reverse primer (10 μM), 2 μl cDNA and 7.2 μl dH_2_O. PCR amplification was performed using the following protocol: 95°C for 1 min, then 40 cycles of 95°C for 15 sec, and finally 60°C for 1 min. The relative abundance of target gene mRNAs was determined from the CT values and plotted as the fold change compared with the control groups. For the PCR analysis, the transcription levels of GAPDH served as a loading control.

### Western blot analysis

Total proteins were extracted using a NP-40 protein extraction kit (cat. no. P0013F), and quantified using an Enhanced BCA Protein Assay kit (cat. no. P0010) (both from Beyotime Institute of Biotechnology, Haimen, China). Protein was transferred onto PVDF membranes by electrophoretic transfer following electrophoretic separation by SDS-PAGE. The membranes were probed with primary antibodies against MMP-2, MMP-9, AKT, p-AKT (1:1,000; Cell Signaling Technology, Beverly, MA, USA), and GAPDH (1:1,000; Santa Cruz Biotechnology) in TBST plus 5% skimmed milk overnight at 4°C. After three washes with TBST, the membranes were incubated with horseradish peroxidase-conjugated secondary antibodies for 1 h at room temperature. Bands were visualized using enhanced chemiluminescence reagents (Thermo Fisher Scientific, Inc., Rockford, IL, USA) and analyzed with a Bio-Rad VersaDoc™ 5000 MP system (Life Science Research, Hercules, CA, USA). GAPDH and AKT were used as loading controls.

### Gelatin zymography of MMP enzyme activity

MMP-2 and MMP-9 activity was measured by SDS-PAGE under non-reducing conditions using gels containing 1% gelatin (Mini-PROTEAN II system; Bio-Rad), and electrophoresis was carried out at 4°C. After washing with 2% Triton X-100 to remove the SDS, the gels were incubated in 37°C with buffer containing 50 mM Tris (pH 7.5), 5 mmol/CaCl_2_ and 1 mmol/l ZnCl_2_ for 18 h. MMP activity was visualized by staining with Coomassie Blue R-250 (Bio-Rad).

### Transwell migration assay

Chemotaxis experiments were performed using a 24-well Transwell chemotaxis chamber technique (Millipore, Billerica, MA, USA) as previously described (21). Briefly, DMEM (600 μl) alone or medium containing 5, 10, 20, 30 and 50 ng/ml recombinant rat SCF (PeproTech, Rocky Hill, NJ, USA) was placed in the lower chamber. A total of 1×10^5^ CSCs in 200 μl of medium were seeded into the upper chamber (pore size, 8 μm). For the inhibition experiment, CSCs were preincubated with a c-Kit blocking antibody or a PI3K/AKT inhibitor, LY294002 (100 nM), for 30 min prior to seeding. The chamber was then incubated for 12 h at 37°C in a humidified atmosphere with 5% CO_2_. The membrane was removed and its upper surface was wiped away with a cotton swab to remove the unmigrated CSCs. The membrane (Millipore) was then fixed in neutral formalin for 10 min at room temperature and then stained with 0.1% crystal violet for 5 min. The number of CSCs that had migrated to the lower surface of the membrane was counted in 10 random high-power fields (HPFs) under a light microscope (Nikon Eclipse 80i; Nikon Instruments, Inc., Melville, NY, USA). A chemotactic index (CI) was calculated to express stimulated migration: CI = stimulated migration (CSCs number per HPF)/random migration (CSCs number per HPF). Each assay was performed in triplicate.

### Statistical analysis

Statistical analysis was performed using PRISM Software^®^ (GraphPad, La Jolla, CA, USA). The data are presented as means ± SD. For analysis of differences between two groups, Student’s t-tests were performed. For multiple groups, ANOVA was carried out followed by the Student-Newman-Keuls method. P<0.05 was considered statistically significant.

## Results

### Characteristics and identification of c-Kit^+^ cells from adult rat hearts

**Figure 1:**
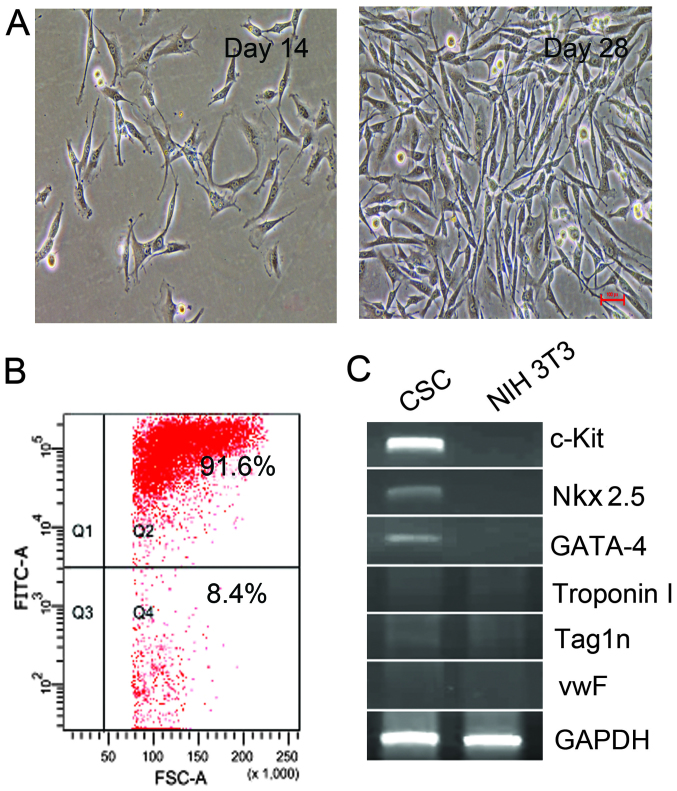
Characteristics and identification of c-Kit^+^ cells from adult rat hearts. (A) Cell morphology for c-Kit^+^ cardiac stem cells (CSCs) on days 14 and 28. Original magnification, ×400. (B) Identification of c-Kit^+^ cells with a purity of 91.6% by flow cytometry. (C) Semi-quantitative RT-PCR analysis of Nkx2.5, GATA-4, Troponin I, vwF and Tagln gene expression in CSCs. NIH 3T3 cells were used as a negative control, and GAPDH as an internal control of PCR.

Using immunomagnetic microbeads, c-Kit^+^ cells were isolated and collected from adult rat hearts. Under light microscopy, the freshly isolated c-Kit^+^ CSCs appeared as small, round and phase-bright cells, and were suspended in the medium. Cells turned into polygonal and spindle-like shape 7 days later, and cells reached confluence at 4 weeks. The purity of c-Kit^+^ CSCs was 91.6% as determined by flow cytometry (Fig. 1B). The expression of c-Kit was detected by RT-qPCR. As shown in Fig. 1C, c-Kit mRNA was detectable in isolated CSCs, while as a negative control, NIH 3T3 cells did not express c-Kit mRNA. Furthermore, Nkx2.5 and GATA-4 mRNA were detectable in the isolated cells, while markers for myocardium (Troponin I), epithelial cells (vwF) and smooth muscle cells (Tag1n) were negative. These results confirmed that the isolated cells were c-Kit^+^ CSCs.

### Effects of SCF on CSC migration in vitro

**Figure 2:**
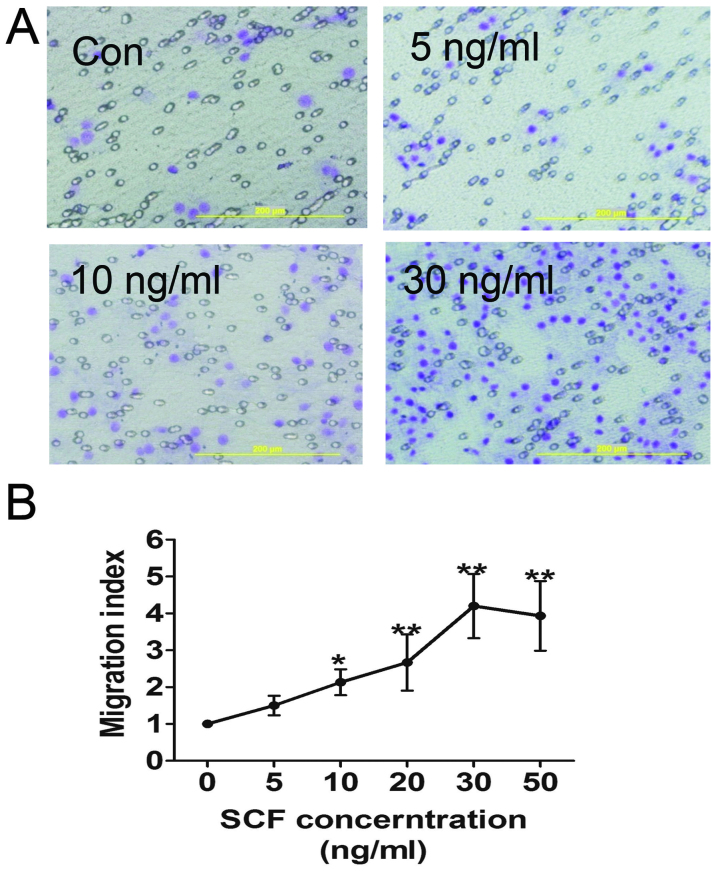
Effect of stem cell factor (SCF) on cardiac stem cell (CSC) migration *in vitro*. (A) Representative images of migrated CSCs treated with varying concentrations of SCF by Transwell-based migration assays. Medium alone was used in the negative control experiments. Original magnification, ×200; bar, 100 μm. (B) SCF-induced CSC migration in a dose-dependent manner. Results are presented as means ± SD. ^*^P<0.05 and ^**^P<0.01 versus control.

A Transwell-based migration assay was established to quantitatively evaluate CSC migration *in vitro*. As shown in Fig. 2, compared with the control group the average number of migrated CSCs increased significantly in the conditioned medium groups with increasing SCF concentration, which reached a peak at 30 ng/ml. Furthermore, SCF-induced CSC migration was inhibited by pre-treatment of the CSCs with SCF antibody, as well as LY294002 (Fig. 4C).

### SCF-mediated MMP-2/-9 expression and enzymatic activity

**Figure 3:**
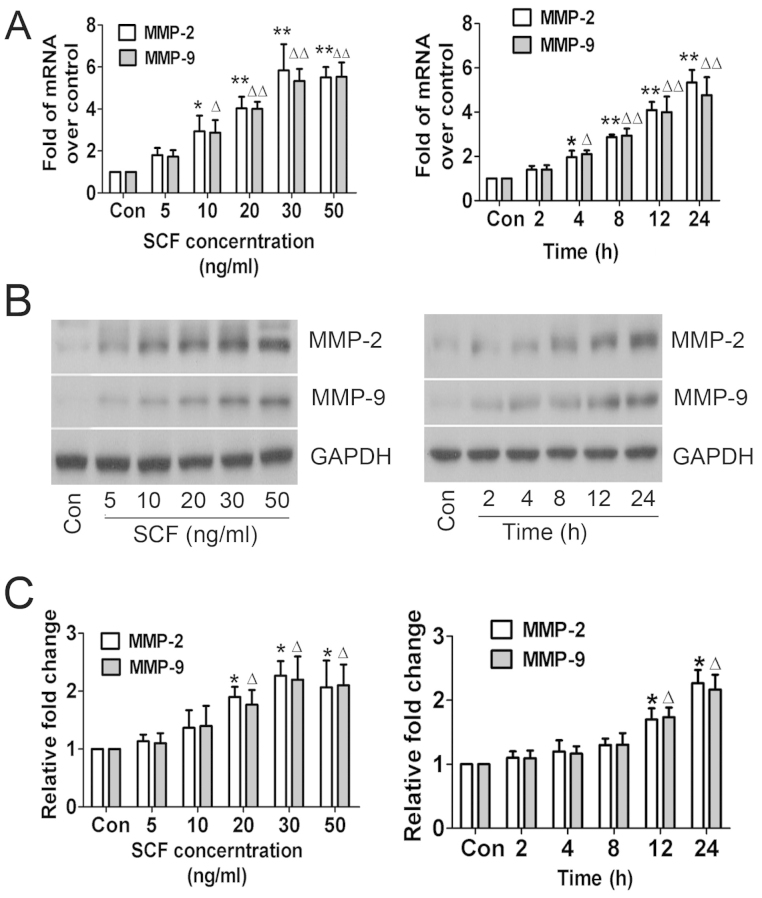
Effect of stem cell factor (SCF) on the expression and activity of matrix metalloproteinase (MMP)-2 and MMP-9. (A) SCF-induced MMP-2 and MMP-9 mRNA expression was measured by quantitative RT-PCR. (B) Western blot analysis of SCF-induced MMP-2 and MMP-9 protein levels. (C) Densitometric analysis of SCF-induced MMP-2 and MMP-9 enzymatic activity. Results are presented as means ± SD. ^*^P<0.05, ^**^P<0.01, ^Δ^P<0.05 and ^ΔΔ^P<0.01 versus control.

The role of SCF in MMP-2 and MMP-9 expression in CSCs *in vitro* was examined. Gelatin zymography assay was conducted based on the theory that MMP-2 and MMP-9 degrades gelatin. The results of the RT-qPCR and western blot analysis revealed that MMP-2 and MMP-9 expression in CSCs was not regulated when treated with higher concentrations of SCF for longer periods of time, which was confirmed by the enzymatic activity results (Fig. 3). The trend of increasing MMP-2 and MMP-9 indicated that SCF mediated their expression and enzymatic activity in a concentration- and time-dependent manner.

### PI3K/AKT is involved in SCF-mediated MMP-2/-9 expression

**Figure 4:**
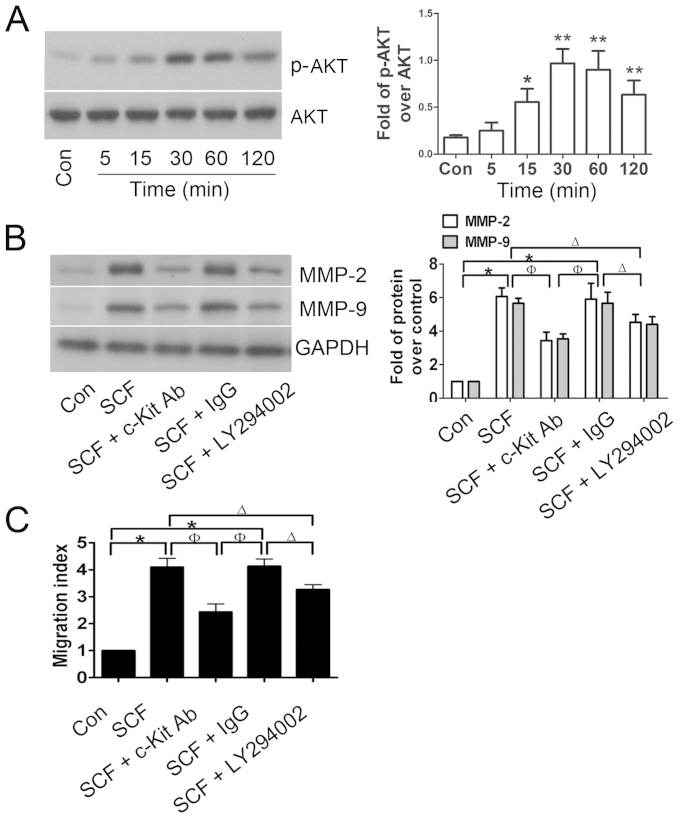
PI3K/AKT involvement in stem cell factor (SCF)-mediated cardiac stem cell (CSC) migration. (A) Western blot analysis of phospho-AKT following SCF stimulation of cardiac stem cells (CSCs) for 0–120 min. (B) CSCs were pre-incubated with SCF antibody (or IgG non-specific antibody) or the PI3K/AKT inhibitor LY294002 for 30 min, and then matrix metalloproteinase (MMP)-2 and MMP-9 protein levels were analyzed by western blot analysis, and (C) the corresponding migration assay was assessed by Transwell experiments. Results are presented as means ± SD. ^*^P<0.05 and ^**^P<0.01 versus control, ^Δ^P<0.05 versus SCF + LY294002, ^Φ^P<0.05 versus SCF + c-Kit Ab.

To explore whether SCF-induced CSC migration was associated with the PI3K/AKT pathway, western blot analysis was performed to detect the expression of total AKT and phospho-AKT protein. As shown in Fig. 4A, total AKT protein expression was not markedly altered, however, the levels of phospho-AKT were significantly increased in SCF-treated CSCs and reached a peak at 30 min after incubation with 30 ng/ml SCF. Furthermore, CSCs were preincubated with c-Kit blocking antibody or LY294002, resulting in significant suppression of the SCF-mediated upregulation of MMP-2 and MMP-9 (Fig. 4B), which was matched with the migration index of the CSCs (Fig. 4C). These results suggested that the PI3K/AKT pathway was involved in SCF-induced CSC migration.

## Discussion

Enzymatic digestion and the tissue expansion method are standard approaches in the isolation of c-Kit^+^ CSCs. Choi *et al* reported that the enzymatic digestion method is more effective in isolating human c-Kit^+^ CSCs compared with the tissue expansion method (26). Using the enzymatic digestion method, the stem cell marker, c-Kit, is efficiently preserved and the isolated CSCs proliferate much better than cells isolated through the tissue expansion method. He *et al* confirmed that MACS following enzymatic digestion is a simple but cost-effective approach that can be used to obtain sufficient numbers of stably-expressed c-Kit^+^ CSCs (27). Furthermore, the differentiation potential of c-Kit^+^ CSCs is preserved after long-term culture for 40 passages (28). In the present investigation, we performed the enzymatic digestion method plus MACS to isolate rat c-Kit^+^ CSCs, as previously described (13,21). As a result, the small, round c-Kit^+^ CSCs were successfully obtained from adult rat hearts. After 4 weeks of culture, cells reached confluence. These c-Kit^+^ CSCs were positive for Nkx2.5 and GATA-4, the markers for early cardiomyocyte commitment, which were in concordance with the results of Choi *et al* (26). However, c-Kit^+^ CSCs derived from newborn hearts were negative for transcription factors and cytoplasmic proteins specific to cardiomycte (Troponin I), smooth muscle cell (Tag1n) and endothelial cell (vwF) and hematopoietic cells (29). Thus, Nkx2.5^+^/GATA-4^+^/c-Kit^+^ CSCs from adult rats may present a relatively late stage of cell differentiation.

Activation of SCF/c-Kit signaling plays a crucial role in a variety of cell biological functions, such as the regulation of cell differentiation and proliferation, cell apoptotic resistance, and mediation of cell migration through the activation of downstream signaling molecules (30). Accumulating data have indicated that the activation of SCF/c-Kit signaling plays a crucial role in mediating stem cell migration and homing. Lutz *et al* demonstrated that local injection of SCF improves myocardial homing of systemically delivered c-Kit^+^ bone marrow-derived stem cells (31), while Kuang *et al* (21) showed that SCF/c-Kit signaling mediated c-Kit^+^ CSC migration via the activation of the downstream p38 cascade. On the other hand, hyperglycemia impairs c-Kit^+^ CSC migration via a reduction in the activity of ERK1/2 and p38 (32), and hyperhomocysteinemia inhibits the homing of CSCs to peri-infarcted areas post-MI in rats with an associated mechanism that may be due to the inhibition of NF-κB (33). Consistent with the above-mentioned reports, in the present investigation we found that c-Kit^+^ CSC chemotaxis was promoted by exogenous SCF, with its CI reaching a peak at a concentration of 30 ng/ml. However, the migratory potential of c-Kit^+^ CSCs promoted by exogenous SCF may be significantly abrogated by SCF antibodies. In future studies, the mechanism of SCF/c-Kit signaling-mediated CSC migration should be investigated.

Considering PI3K/AKT signaling is important for cell survival, apoptosis and motility (3), we suggest that PI3K/AKT singling is involved in the process of SCF/c-Kit signaling-mediated CSC migration. The current results are consistent with our hypothesis in that administration of the PI3K/AKT inhibitor LY294002 clearly suppressed SCF-mediated CSC migration. Our results suggest that there is a connection between SCF/c-Kit and PI3K/AKT signaling. A previous report indicated that activation of the PI3K/AKT pathway is crucial for VEGF-mediated c-Kit^+^ CSC migration *in vitro* and *in vivo* (22). Furthermore, PI3K/AKT signaling is important for infused stem cell survival post-MI (23–25). Thus, this pathway plays a clear role in the biology of c-Kit^+^ CSCs, and deserves additional attention in the field of MI cellular therapy based on stem cell transplantation.

Cell migration is a complex and elaborately regulated process. Several factors such as adhesion strength and the type of substratum [including extracellular matrix (ECM) ligands], external migratory signals and cues, mechanical pliability, dimensionality, and the organization of the cellular cytoskeleton determine the specifics of cellular motility (34,35). Among these, the complex interactions of cells with ECM are crucial in mediating and regulating cell migration (36). As a critical factor mediating the interactions of cells with the ECM, the abnormal expression of MMPs is thought to be an important determinant in cell migration, and there is considerable evidence that this occurs in cancerous cells. For instance, the upregulation of MMP-2 and MMP-9 collagenases is associated with the invasion and metastasis of cervical uterine neoplasm (37). Consistent with the results obtained in cancer, the upregulation of MMP-2 and MMP-9 also contributes to the migratory/invasive behavior of human mesenchymal stem cells (hMSC) (38). Furthermore, activation of PI3K/AKT signaling is sufficient to induce MMP-2 and MMP-9 expression in erythropoietin-treated mouse brain endothelial cells (39). Therefore, we conducted experiments to investigate whether a high expression of MMP-2 and MMP-9 contributed to SCF-enhanced CSC motility. As expected, treatment of the c-Kit^+^ CSCs with exogenous SCF effectively activated PI3K/AKT signaling and led to the upregulation of MMP-2 and MMP-9 expression and activity in a concentration- and time-dependent manner. We observed that c-Kit blocking antibody or LY294002 significantly attenuated SCF-induced MMP-2 and MMP-9 expression, which was accompanied by reduced CSC CI values. Thus, it seems that activation of the SCF/c-Kit/PI3K/AKT/MMP-2/-9 pathway is critical for CSC migration, which is consistent with the rationale that hepatocyte growth factor (HGF) increases the function of MMP-2 and MMP-9 in CSCs, thus facilitating their migration (40).

Taken together, our current study provides evidence that SCF partially mediated c-Kit^+^ CSC migration via the activation of PI3K/AKTMMP-2/-9 signaling. A correlation between SCF/c-Kit and PI3K/AKT signaling was identified, which explains some of the diverse biological activity of CSCs. In conclusion, these findings may assist in the progression of translational medicine that utilizes CSCs in the repair of injured heart tissue.

## Figures and Tables

**Table I:** Primers used for PCR.

Genes (ID)	Semi-quantitative PCR sequences (5′→3′)
C-Kit (D12524.1)	F: ttggcaaagaagacaacgac R: gcacagacaccactgggata
GATA-4 (NM_144730.1)	F: gcagaaacaacaaagggaaat R: gggagaaacagcgtaaatga
Nkx2.5 (NM_053651.1)	F: ttccagaaccgccgctacaag R: ccgacgccaaagttcacgaag
Troponin I (NM_017184.1)	F: gggagactggaggaagaacg R: gagggaacaacaacagcaaaa
vwF (NM_053889.1)	F: cctacggcttgcacgattca R: ccacttcctcttccgacttac
Tagln (NM_031549)	F: gaggactgtaatggctttgg R: gccttccctttctaactgat
GAPDH (BC059110)	F: cagtgccagcctcgtctcat R: ggggccatccacagtcttc

Genes (ID)	Quantitative PCR sequences (5′→3′)

MMP-2 (NM_031054)	F: ggaagcatcaaatcggactg R: caccctcttaaatctgaaatcacc
MMP-9 (NM_031055)	F: cccacttactttggaaacg R: gaagatgaatggaaatacgc
GAPDH (BC059110)	F: cccatctatgagggttacgc R: tttaatgtcacgcacgatttc
